# Improving Balance Between Oxygen Permeability and Stability of Ba_0.5_Sr_0.5_Co_0.8_Fe_0.2_O_3−_*_δ_* Through High-Entropy Design

**DOI:** 10.3390/membranes15080232

**Published:** 2025-08-01

**Authors:** Yongfan Zhu, Meng Wu, Guangru Zhang, Zhengkun Liu, Gongping Liu

**Affiliations:** 1 State Key Laboratory of Materials-Oriented Chemical Engineering, College of Chemical Engineering, Nanjing Tech University, No. 30 Puzhu Road (S), Nanjing 211816, China; yongfanzhu@njtech.edu.cn (Y.Z.); guangru.zhang@njtech.edu.cn (G.Z.);; 2 Nanjing Tech University Suzhou Future Membrane Technology Innovation Center, Suzhou 215300, China; 3 Quzhou Membrane Material Innovation Institute, Nanjing Tech University Quzhou Base, 99 Zheda Rd, Quzhou 324000, China

**Keywords:** high-entropy design, perovskite oxide, oxygen permeation, CO_2_ resistance

## Abstract

Currently, the trade-off between oxygen permeation flux and structural stability in conventional perovskite oxides restricts the practical application of oxygen permeable membranes. In this study, a high-entropy design was applied to the B-site of BSCF matrix materials, resulting in the successful synthesis of a high-entropy perovskite, Ba_0.5_Sr_0.5_Co_0.71_Fe_0.2_Ta_0.03_Ni_0.03_Zr_0.03_O_3−*δ*_. The crystal structure, microstructure, and elemental composition of the material were systematically characterized and analyzed. Theoretical analysis and experimental characterization confirm that the material exhibits a stable single-phase high-entropy perovskite oxide structure. Under He as the sweep gas, the membrane achieved an oxygen permeation flux of 1.28 mL·cm^−2^·min^−1^ and operated stably for over 100 h (1 mm thick, 900 °C). In a 20% CO_2_/He atmosphere, the flux remained above 0.92 mL·cm^−2^·min^−1^ for over 100 h, demonstrating good CO_2_ tolerance. Notably, when the sweep gas is returned to the pure He atmosphere, the oxygen permeation flux fully recovers to 1.28 mL·cm^−2^·min^−1^, with no evidence of leakage. These findings indicate that the proposed B-site doping strategy can break the trade-off between oxygen permeability and structural stability in conventional perovskite membranes. This advancement supports the industrialization of oxygen permeable membranes and offers valuable theoretical guidance for the design of high-performance perovskite materials.

## 1. Introduction

In recent years, perovskite mixed conductor materials have shown great potential in high-purity gas production [1,2,3], energy development [4], and pollution control [5,6]. The perovskite mixed ionic electronic conductor membranes offer highly efficient and high-purity oxygen production, and can be easily integrated with large-scale devices, such as nitrogen-free and oxygen-enriched combustion [7,8,9]. As research on perovskite oxygen permeable membrane materials has progressed, their potential for industrial applications has become increasingly apparent. Shao et al. [10] found that among Ba_1−_*_x_*Sr*_x_*Co_1−_*_y_*Fe*_y_*O_3−_*_δ_* materials, Ba_0.5_Sr_0.5_Co_0.8_Fe_0.2_O_3−_*_δ_* (BSCF) demonstrated the highest oxygen permeation flux and stability, meeting the performance criteria for industrial use. However, maintaining stability in a CO_2_ atmosphere poses a significant challenge for BSCF oxygen permeable membranes. Zhang et al. [11] developed a series of SrFe_0_._8_*X*_0.2_O_3−*δ*_ (*X* = Zr, Mo, and W) materials that exhibit excellent stability in both air and CO_2_ atmospheres. However, their poor oxygen permeability limits their commercial application prospects. Therefore, achieving a balance between oxygen permeability and stability poses CO_2_ atmospheres is crucial for perovskite oxygen-permeable membranes.

Nowadays, optimizing the oxygen permeability and stability of membrane materials involves metal element doping/substitution [12,13], the construction of A/B-site defects [14,15,16], and non-metal element doping [3,17,18]. Most alkaline earth, rare earth, and transition metals are capable of forming perovskite oxides, which result in special physical and chemical properties [19,20]. Metal doping/substitution has always been a significant approach for developing high-performance perovskite oxygen permeable membranes. Over the past two decades, metal doping/substitution has primarily focused on single-element modifications, such as Ba_0.5_Sr_0.5_Co_0.7_Fe_0.2_Ni_0.1_O_3−*δ*_ [12], Ba_0.5_Sr_0.5_Co_0.78_Fe_0.2_W_0.02_O_3−*δ*_ [21], Ba_0.5_Sr_0.5_Co_0.75_Mo_0.05_ Fe_0.2_O_3−*δ*_ [22], etc. However, this strategy may not be sufficient to balance both oxygen permeability and stability. Therefore, exploring advanced metal doping strategies to design and develop perovskite oxygen permeable membrane materials with better performance is necessary.

**Figure 1:**
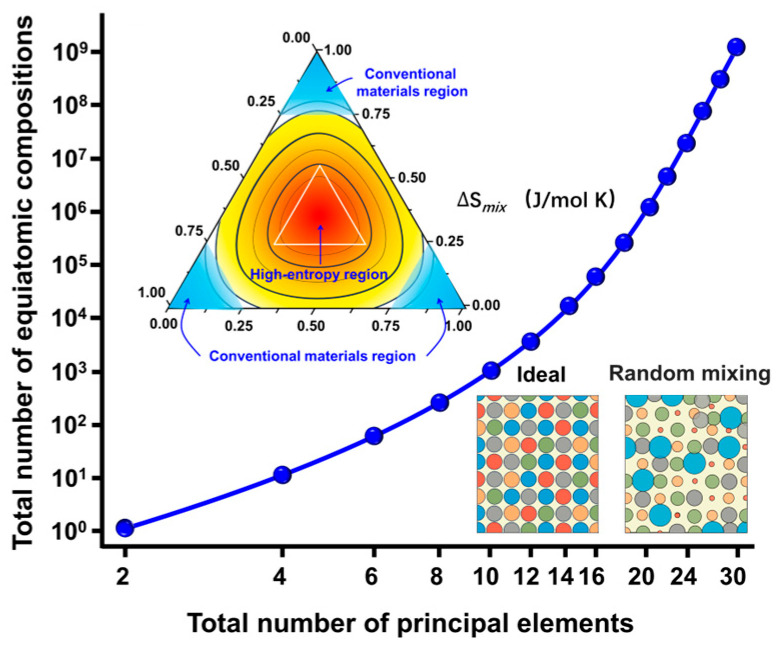
The scheme of the high-entropy design idea. Reproduced with permission from [29]. Copyright (2016) Elsevier.

In contrast to the conventional doping/substitution strategies, the high-entropy perovskite oxide design involves at least five or more elements in the A- or B-site (Figure 1), with a configurational entropy greater than 1.5*R* [23,24] (*R* = 8.314 J·K^−1^·mol^−1^). In 2018, Jiang et al. [24] were the first to synthesize and report high-entropy perovskite oxide materials. Subsequently, Mattia et al. [25] successfully fabricated dense high-entropy perovskite oxide ceramics. High-entropy perovskite oxide membranes have garnered significant attention from materials scientists due to their unique properties and promising practical applications, such as Wang et al. [26,27], who developed BSCF-based high-entropy perovskite materials (doped elements: Ca, La, Gd, Bi, Zr, Ni, Cu, Al) that exhibit good performance at medium and low temperatures. Zhao et al. [28] developed Pr_1−_*_x_*Sr*_x_* (Cr, Mn, Fe, Co, Ni) O_3−_*_δ_* (*x* = 0–0.5) materials with enhanced stability in CO_2_ environments. Our previous research also produced La_0.2−_*_x_*Pr_0.2_Nd_0.2_Ba_0.2_Sr_0.2_Co_0.8−_*_y_*Fe_0.2_Ni*_y_*O_3−*δ*_ (*x* = 0–0.1, *y* = 0–0.1) materials that remain stable under CO_2_ environments over 120 h [14]. Although these materials exhibit high stability in CO_2_ environments, their low oxygen permeation flux still limits their further practical applications.

In this work, we applied a high-entropy design strategy to the B-site of BSCF by incorporating Ta, Zr, and Ni. The high oxide formation and sublimation enthalpies of Ta and Zr enhance the metal–oxygen bond energy, thereby improving the CO_2_ resistance of the perovskite structure [30,31]. Ni, known for its variable valence states, was introduced to enhance oxygen permeability, which may be reduced by the incorporation of Ta and Zr [12]. As a result, the high-entropy perovskite material Ba_0.5_Sr_0.5_Co_0.71_Fe_0.2_Ta_0.03_Zr_0.03_Ni_0.03_ O_3−δ_ (BSCFTZN) was successfully synthesized and its physicochemical properties were systematically investigated. Oxygen permeability tests revealed that the BSCFTZ membrane exhibited high oxygen flux, strong CO_2_ tolerance, and excellent long-term stability. Under a helium atmosphere, the oxygen flux remained stable at 1.28 mL·min^−1^·cm^−2^. In a 20% CO_2_ environment, the flux remained above 0.92 mL·min^−1^·cm^−2^ for over 100 h. Upon switching back to a helium atmosphere, the oxygen flux fully recovered to its initial value, with no detectable leakage. These results demonstrate the outstanding stability of the BSCFTZ material. Notably, the combination of high oxygen permeation performance and strong stability demonstrates that high-entropy design can achieve a favorable balance between oxygen flux and structural stability in oxygen-permeable membranes.

## 2. Materials and Methods

### 2.1. Synthesis of Materials

**Figure 2:**
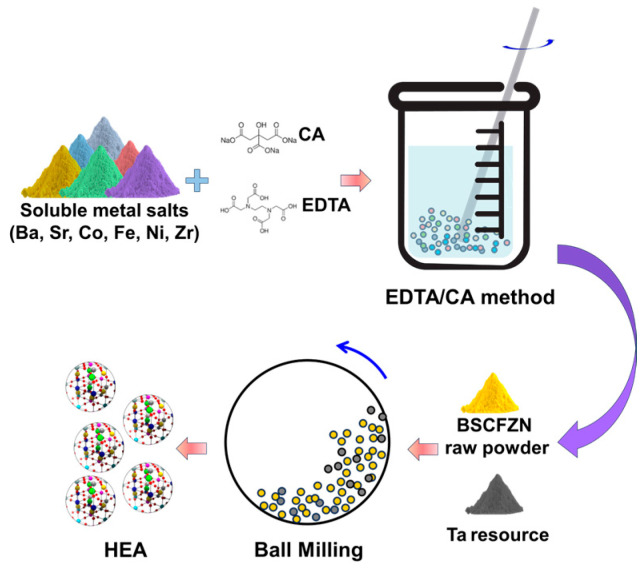
The scheme of the fabrication of BSCFTZN high-entropy perovskite.

The BSCFTZN perovskite was synthesized using the EDTA citric acid (CA) and solid phase reaction methods. The reagents are listed in Table 1. The soluble metal salts were weighed according to the stoichiometric ratio and dissolved in a small amount of deionized water under stirring (room temperature) until a clear solution was obtained. EDTA and CA were used as complexants in a molar ratio of 1:1:2 (total metal ions:EDTA:CA). Ammonia solution (NH_3_·H_2_O) was added as a pH adjuster to maintain the pH between 7.0 and 7.2 during the preparation process. The BSCFZN raw material powder was obtained by heating at 250 °C for 8 h. The powder and Ta_2_O_5_ were mixed in a stoichiometric ratio using ethanol as the solvent and ball-milled for 48 h at 285 RPM (QM-3SP2, Nanjing Nanda Instrument Co., Ltd., Nanjing, China). The mixture was subsequently dried, sieved, and calcined at 900 °C to obtain the BSCFTZN powder (Figure 2).

### 2.2. Membrane Testing

Membrane precursors were formed by isostatic pressing at 15 MPa using Polyvinyl alcohol (PVA) as a binder. The membrane precursors were then sintered at 1050 °C with a heating rate of 2 °C·min^−1^ and held at the target temperature for 300 min. After the sintering and air-tightness test, the dense membranes were polished to a thickness of 1 mm. Oxygen permeation tests were conducted using homemade equipment, as described in our previous work [3]. During testing, the oxygen flux was analyzed using a gas chromatograph (GC-7820A, Agilent, Santa Clara, CA, USA) equipped with a 5 Å molecular sieve column. The tests were conducted over a temperature range of 650–900 °C, using ambient air as the feed gas and helium as the purge gas (60–90 mL·min^−1^). The densities of the sintered membranes were measured using the Archimedes method with deionized water at 25 °C. Only membranes with a relative density exceeding 90% were selected for oxygen permeation testing. The theoretical density of BSCFTZC powder was calculated to be 5.5261 g·cm^−3^ based on its lattice constants at room temperature. The oxygen leakage concentration on the sweep side remained below 0.5%.

### 2.3. Characterization

The crystal structures of the BSCFTZN powders and membranes were observed using X-ray diffraction (XRD, Rigaku Smart Lab 9KW, Tokyo, Japan) with a Cu Kα radiation source (1.5418 Å). A nickel filter was used to suppress the Cu Kβ radiation. The contribution of Cu Kα_2_ radiation was not subtracted. The measurements were performed with a step size of 0.02° and a 2°·min^−1^ scanning rate. The XRD patterns were analyzed and refined using Jadex-ICDD V 9.1 software. The accuracy of the whole patterns fitting (WPF) and Rietveld refinement was confirmed by an R-factor below 10% and an R/E ratio below 2.5. The membrane microstructures were examined using a field emission scanning electron microscope with an acceleration voltage of 10 kV (FE-SEM, Phenom Pharos G2,Guangdong, China). Elemental distribution was analyzed using an Energy Dispersive Spectrometer (EDS, AztecLiveOne, Oxford, UK). The accelerating voltage was set to 15 kV and the measurements were conducted under high vacuum conditions. The thermal expansion of the materials was measured using a dilatometer (Netzsch DIL 402, Waldkraiburg, Germany). Samples were heated to 900 °C at a rate of 5 °C·min^−1^ in an atmosphere of nitrogen, air, and carbon dioxide, with measurements being continuously recorded.

### 2.4. Formatting of Mathematical Components

The Goldschmidt tolerance factor (*t*), the size difference of the cations at the A-site (*δ*(*R*_*A*_)) and B-site (*δ*(*R_B_*)), are used to determine the structural stability of the target perovskite oxide [32]. In addition, in high-entropy perovskites, the configurational entropy must exceed 1.5 *R* [24]. These parameters are calculated using the following equations:

(1) $$t=\frac{r_{A}+r_{O}}{\sqrt{2}\left(r_{B}+r_{O}\right)}$$

where $r_{A}$, $r_{B}$, and $r_{O}$ are the average ionic radii of *A*, *B* sites, and oxygen, respectively.

(2) $$\delta \left(R_{A}\right)=\sqrt{\sum_{i=1}^{N}c_{i}{\left(1-\frac{R_{A_{i}}}{\left(\sum_{i=1}^{N}c_{i}R_{A_{i}}\right)}\right)}^{2}}$$

where $R_{A_{i}}$ and $c_{i}$ are the cation radius and corresponding molar fraction of the *A*-site element, respectively.

(3) $$\delta \left(R_{B}\right)=\sqrt{\sum_{i=1}^{N}c_{i}{\left(1-\frac{R_{B_{i}}}{\left(\sum_{i=1}^{N}c_{i}R_{B_{i}}\right)}\right)}^{2}}$$

where $R_{B_{i}}$ and $c_{i}$ are the cation radius and corresponding molar fraction of the *B* site element, respectively.

(4) $${\Delta S}_{mix}=-R\left[{\left(\sum_{a=1}^{n}x_{a}\;lnx_{a}\right)}_{A-site}+{\left(\sum_{b=1}^{n}x_{b}\;lnx_{b}\right)}_{B-site}+3{\left(\sum_{c=1}^{n}x_{c}\;lnx_{c}\right)}_{O-site}\right]$$

where $x_{a}$, $x_{b}$, and $x_{c}$ are the molar fractions of metal ions at the *A*, *B*, and oxygen sites, respectively.

## 3. Results and Discussion

### 3.1. Material Design

**Figure A1:**
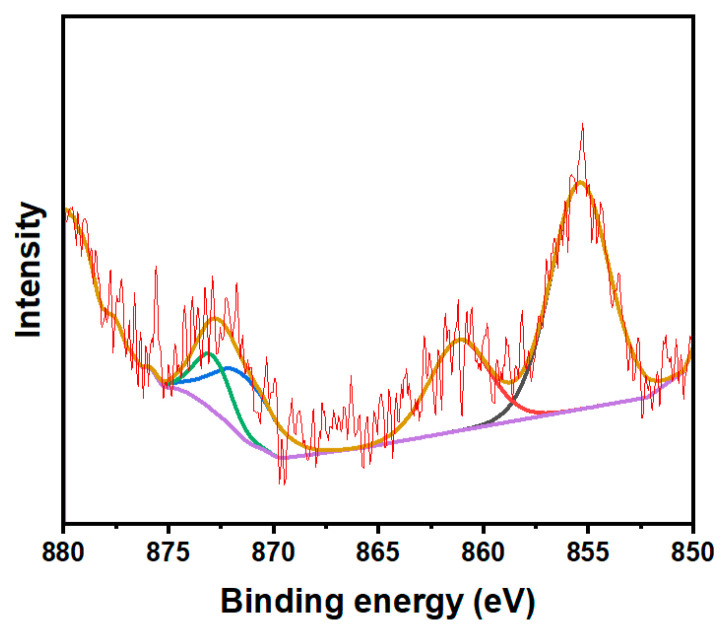
Ni2p XPS spectra of BSCFTZN.

In this work, Ta, Ni, and Zr were introduced into the B-site of BSCF to develop a high-entropy doping material. Specifically, the high valence state of Ta^5+^ enhances the resistance of the material to CO_2_ [33]. Zr^4+^ contributes chemical and thermal stability, suppressing phase transitions and grain growth at elevated temperatures, thus improving long-term stability in CO_2_-rich environments [25]. The variable valence of Ni^2+^/Ni^3+^ enhances electronic conductivity (Figure A1) and its moderate ionic radius helps preserve the integrity of the perovskite structure [12]. Notably, while Ta and Zr doping slightly reduces oxygen vacancy concentration, incorporating Ni compensates for this effect, enabling the material to retain high oxygen ion conductivity.

The calculation of four parameters is shown in Table 2. The results indicate that the Goldschmidt tolerance factor (*t*) of BSCFTZN is 1.00, aligning closely with the theoretical value for an ideal cubic perovskite structure. Additionally, the small *δ*(*R_A_*) and *δ*(*R_B_*) values favor the formation of a single-phase structure through cation incorporation into the perovskite lattice. Furthermore, the configurational entropy (Δ*S_mix_*) exceeds 1.5 *R* (*R* = 8.314 J·mol^−1^·K^−1^), satisfying the requirement for high-entropy perovskite formation.

### 3.2. Crystal Phase and Microstructure

**Figure 3:**
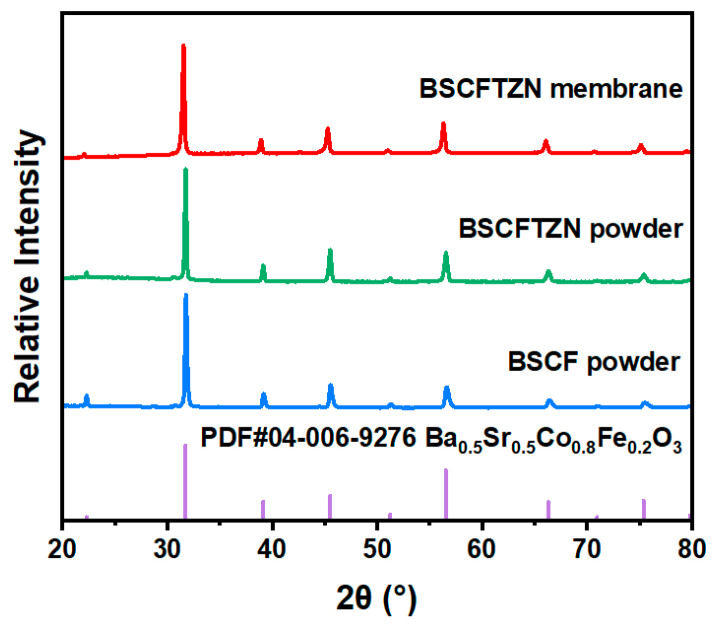
XRD patterns of the BSCF powder, BSCFTZN powder, and BSCFTZN membrane.

**Figure 4:**
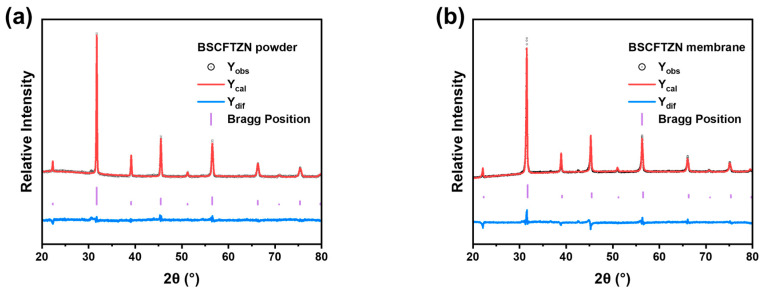
The refined XRD data (**a**) BSCFTZN powder and (**b**) BSCFTZN membrane.

Figure 3 presents the XRD patterns of BSCFTZN and BSCF powders (900 °C), along with the BSCFTZN membrane (1050 °C). The results confirm that both BSCFTZN and BSCF powders exhibit a cubic perovskite structure; the same structure is also observed in the BSCFTZN membrane. Rietveld refinement results (Figure 4) and the lower oxygen content (Table A1) indicate that both BSCFTZN powder and membrane exhibit a single-phase cubic perovskite structure (Pm-3m, a = b = c = 3.99 Å, R = 4.44% E = 2.19% R/E = 2.03 for BSCFTZN powder, R = 5.13% E = 2.08% R/E = 2.47 for BSCFTZN membrane).

**Figure 5:**
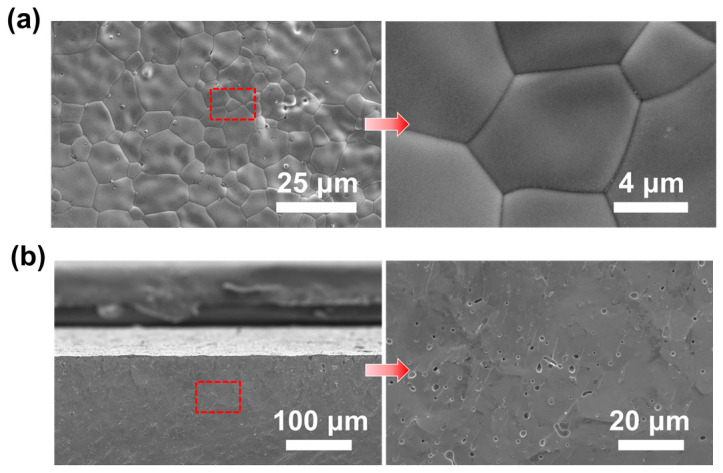
SEM images of BSCFTZN membrane (**a**) surface and local enlarged view, (**b**) cross-section and local enlarged view.

The microstructure of the BSCFTZN disk membrane was examined using an FE-SEM, as shown in Figure 5. The surface images in Figure 5a reveal a dense membrane structure with well-defined grain boundaries, no secondary phases, and grain sizes primarily between 5 and 20 μm, with no through-pores or cracks. The membrane exhibited a relative density of 97.8%, indicating its suitability for oxygen permeation testing. In addition, cross-sectional images in Figure 5b further confirm the dense structure of the membrane, with no through-pores observed. This dense microstructure enhances the mechanical strength of the membrane and reduces the risk of breakage during oxygen permeation operation.

**Figure 6:**
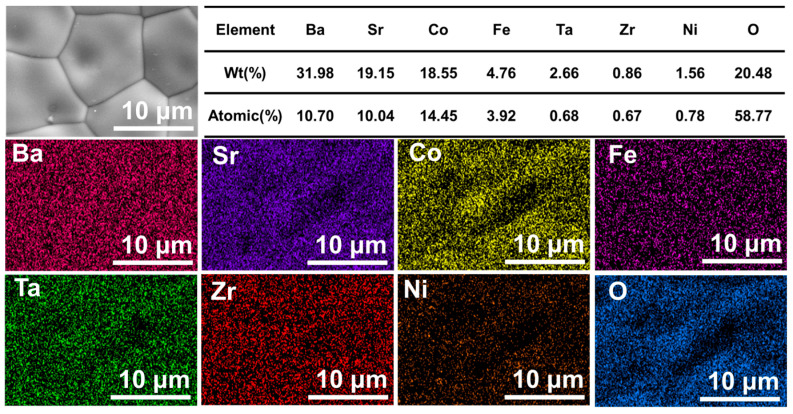
EDS mapping data of the surface for the BSCFTZN membrane.

To further confirm the successful incorporation of Ta, Ni, and Zr into the BSCF matrix, the surface of the BSCFTZN perovskite membrane sintered at 1050 °C was analyzed using an EDS, as shown in Figure 6. The EDS mapping data confirm the uniform distribution of Ba, Sr, Co, Fe, Ta, Zr, Ni, and O across the membrane surface, with no evidence of localized enrichment or segregation. This result also indicates that Ta, Ni, and Zr are effectively incorporated into the perovskite lattice rather than accumulating on the surface or forming secondary phases. However, there are some errors in the quantitative analysis of EDS. Quantitative EDS analysis reveals atomic percentages, further confirming the doping efficiency and elemental homogeneity (Table A2).

### 3.3. Thermal Expansion Behavior Analysis

**Figure 7:**
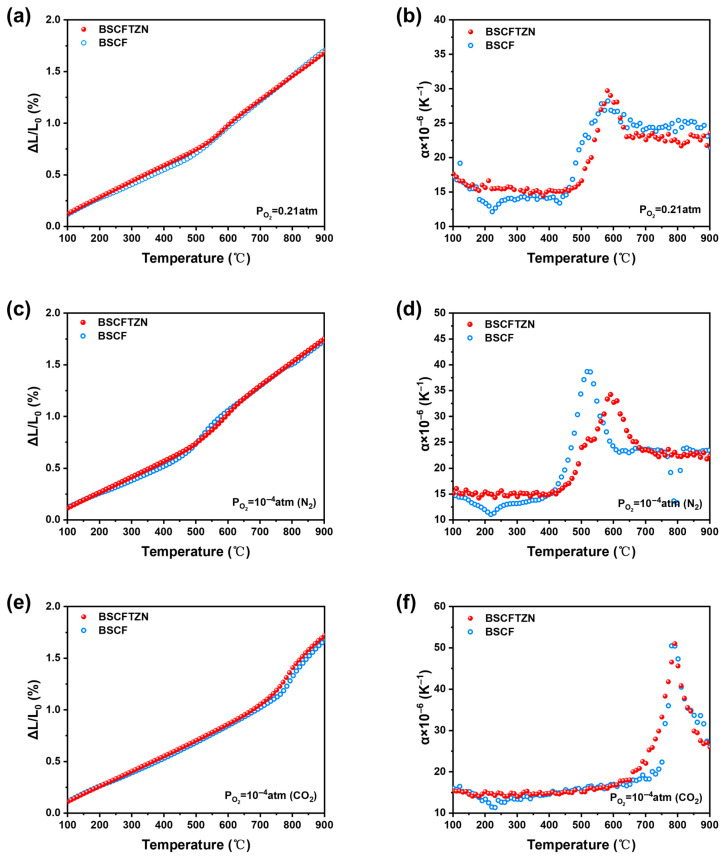
Thermal expansion behavior and thermal expansion coefficients of BSCFTZN and BSCF samples in air (**a**,**b**), nitrogen (**c**,**d**), and carbon dioxide (**e**,**f**) atmospheres.

Figure 7 systematically compares the thermal expansion behavior of BSCFTZN and BSCF strip membranes (6 × 6 × 20 mm) under various atmospheres. In air (Figure 7a,b), both materials exhibit similar overall thermal expansion curves. However, the smoother thermal expansion coefficient curve of BSCFTZN indicates enhanced thermal stability due to the high-entropy design. In a nitrogen atmosphere (Figure 7c,d), BSCF displays a higher expansion rate than BSCFTZN between 400 and 900 °C. Under a CO_2_ atmosphere (Figure 7e,f), BSCFTZN and BSCF exhibit comparable thermal expansion coefficients within 600–900 °C. However, BSCFTZN demonstrates a more stable and continuous curve. The low chemical expansion of BSCFTZN effectively prolongs membrane lifespan, reduces thermal stress during operation, and enhances sealing performance. These advantages support consistent and controllable behavior during large-scale modular integration, facilitating industrial implementation. The oxygen permeability corroborates these results, as shown in Figure 9, where BSCFTZN maintains a flux of 0.38 mL·cm^−2^·min^−1^ in pure CO_2_, whereas the flux of BSCF drops to nearly zero. These findings demonstrate that the dynamic stability imparted by high-entropy design—driven by multi-element synergy and a configurational entropy (Δ*S_mix_*) greater than 1.5R—not only enhances material stability under harsh conditions but also preserves functional performance.

### 3.4. Oxygen Permeation Test

**Figure 8:**
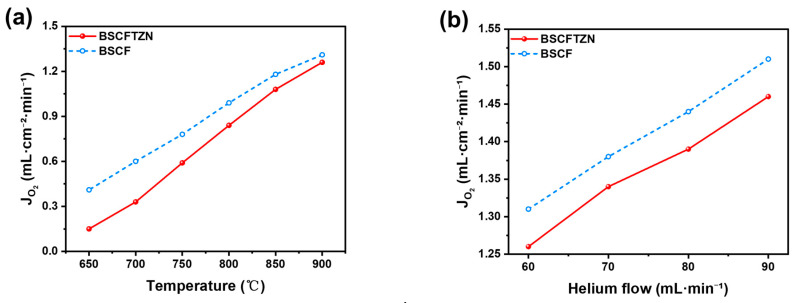
Oxygen permeation flux of BSCFTZN and BSCF membrane (**a**) function of temperature (ambient air, He flow rate: 60 mL·min^−1^); (**b**) function of sweep gas flow rate (900 °C, ambient air).

The oxygen permeation performance was evaluated using a custom-built laboratory test apparatus, with the results presented in Figure 8. The oxygen permeation flux increases significantly with rising temperature. At 900 °C, the BSCFTZN membrane achieves a flux of 1.28 mL·cm^−2^·min^−1^, slightly lower than that of the BSCF membrane (1.36 mL·cm^−2^·min^−1^). This is because the high oxidation states of Ta^5+^ and Zr^4+^ limit the variable valence ability at the B-site in the BSCF structure material and reduce the oxygen vacancy concentration (Table A1) [33]. The synergistic doping of Ni can support an additional oxide valence state variation possibility, optimize the material (variable valence characteristics, Ni^2+^/Ni^3+^) [12,14], and effectively compensate for the reduction in oxygen vacancy concentration introduced by Ta and Zr. The measured activation energies for oxygen permeation also suggest that the BSCF membrane possesses higher oxygen flux compared to the BSCFTZN membrane. The temperature-dependent difference in activation energy is attributed to changes in the crystal structure of the perovskite oxide. Figure 8b shows that with the increase of the sweep gas flow rate, the oxygen permeation flux of the membrane is significantly increased. This is attributed to the enhancement of mass exchange on the membrane surface. A higher sweep gas flow rate can help transport the oxygen faster away from the sweep side, thereby forcing the equilibrium to deliver more oxygen [34].

**Figure 9:**
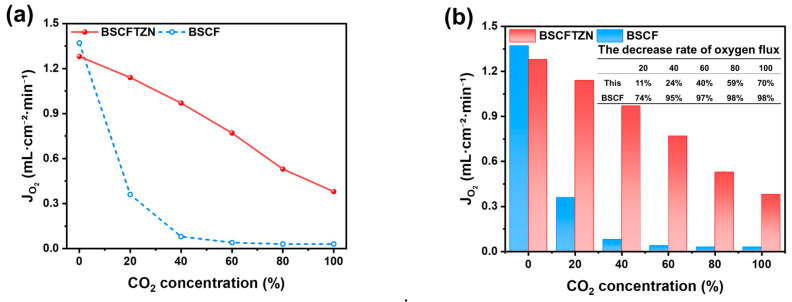
Oxygen permeation flux of BSCFTZN and BSCF (**a**) dependent on CO_2_ concentration, (**b**) decreases with value.

Figure 9 illustrates the variation in oxygen permeation flux for BSCFTZN and BSCF membranes under different CO_2_ concentrations in the sweep gas. A CO_2_/He mixture was used as the sweep gas (60 mL·min^−1^) at 900 °C, with CO_2_ concentrations ranging from 0% to 100%. The two membranes exhibited distinct declines in oxygen permeation flux as CO_2_ concentration increased. Switching the sweep gas from pure He to a 20% CO_2_/He mixture led to a sharp 73.7% decline in oxygen permeation for the BSCF membrane, whereas the BSCFTZN membrane exhibited only a 10.9% reduction. Under pure CO_2_ conditions, the BSCFTZN membrane maintained an oxygen permeation flux of 0.38 mL·cm^−2^·min^−1^, while the oxygen flux of the BSCF membrane dropped to nearly zero. This performance offers significant advantages over conventional perovskite membranes (Table 3). This significant difference demonstrates that the high-entropy configuration of the BSCFTZN material enhances its CO_2_ resistance, extending membrane durability. These findings confirm that high-entropy design is an effective strategy for improving material stability under demanding operating conditions.

### 3.5. Long-Term Test

**Figure 10:**
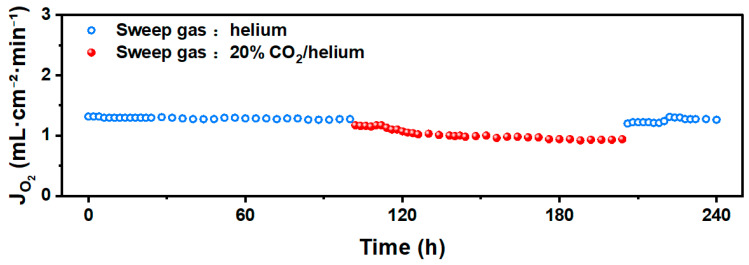
Long-term test of BSCFTZN membrane.

**Figure 11:**
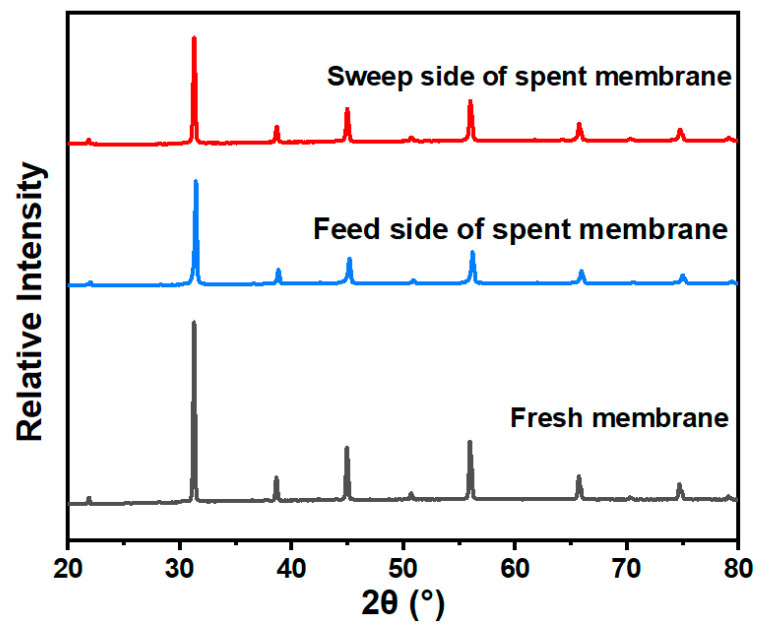
XRD patterns of the BSCFTZN membrane after the long-term test.

**Figure 12:**
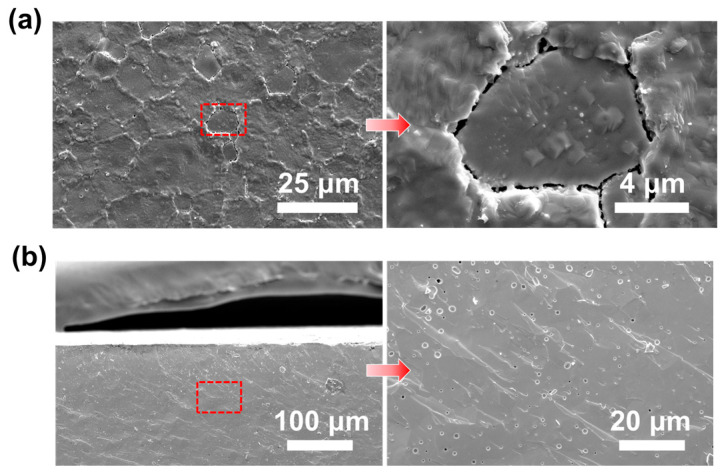
SEM images of BSCFTZN membrane after long-term test (**a**) surface and local enlarged view, (**b**) cross-section and local enlarged view.

Figure 10 presents the long-term oxygen permeation test results for the BSCFTZN membrane (1 mm thickness, ambient air, 900 °C). Under a He atmosphere (60 mL·min^−1^), the BSCFTZN membrane demonstrated excellent stability, maintaining an oxygen permeation flux of approximately 1.28 mL·cm^−2^·min^−1^ with no significant fluctuations or anomalies over 100 h of continuous operation. To assess the impact of CO_2_ on membrane performance, a 20% CO_2_/He mixture (60 mL·min^−1^) was used as the sweep gas for oxygen permeation testing. Under these conditions, the membrane exhibited strong CO_2_ tolerance, and the oxygen permeation flux remained stable at 0.92 mL·cm^−2^·min^−1^ for 100 h. Furthermore, by switching the sweep gas back to pure He, the oxygen permeation flux can immediately return to 1.28 mL·cm^−2^·min^−1^, indicating no irreversible structural changes. This finding aligns with the XRD analysis shown in Figure 11. Figure 12 presents the post-test images of the BSCFTZN membrane after the long-term test. The grain boundaries on the membrane surface appear blurred; this is due to extended oxygen permeation and exposure to CO_2_. As shown in Figure 12b, the cross-sectional images confirm that the BSCFTZN membrane remains intact and maintains a dense structure. These results confirm that the BSCFTZN membrane offers outstanding stability and CO_2_ resistance, making it a strong candidate for oxygen separation in CO_2_-rich environments and related applications.

## 4. Conclusions

In this study, a high-entropy design was applied to the B-site of BSCF by incorporating Ta, Zr, and Ni, resulting in the successful synthesis of the single-phase high-entropy perovskite oxide Ba_0.5_Sr_0.5_Co_0.71_Fe_0.2_Ta_0.03_Zr_0.03_Ni_0.03_O_3−_*_δ_* material. The oxygen permeability of the BSCFTZN membrane was evaluated under varying CO_2_ concentrations, along with long-term stability testing. Experimental results demonstrate that the BSCFTZN membrane can maintain an oxygen permeation flux of 0.92 mL·cm^−2^·min^−1^ over 100 h in a 20% CO_2_ atmosphere. This performance overcomes the long-standing trade-off between high oxygen permeability and structural stability in perovskite membranes. This offers new insights into the design of perovskite-based oxygen permeable membranes and provides an impetus for industrial development.

## Figures and Tables

**Table 1:** The reagent specification and manufacturers in experiments.

Reagent	Purity	Manufacturer
Ba(NO_3_)_2_	99.999%	Macklin, Shanghai, China
Sr(NO_3_)_2_,	99.999%	Macklin, Shanghai, China
Co(NO_3_)_2_·6H_2_O	99.999%	Macklin, Shanghai, China
Fe(NO_3_)_2_·9H_2_O	99.999%	Macklin, Shanghai, China
ZrO(NO_3_)_2_·xH_2_O	99.999%	Macklin, Shanghai, China
Ni(NO_3_)_2_·6H_2_O	99.999%	Macklin, Shanghai, China
Ta_2_O_5_	99%	Sinopharm Chemical Reagent Co., Ltd. Sunzhou, China

**Table 2:** Four parameters of the BSCFTZN.

Materials	*t*	*δ*(*R_A_*)	*δ*(*R_B_*)	Δ*S_mix_*
BSCFTZN	1.0	5.11%	2.99%	1.57R

**Table 3:** Comparison of oxygen flux between the BSCFTNZ membrane and other perovskite membranes.

Materials	Thickness (mm)	Temperature (°C)	$J_{O_{2}}$ (mL·cm^−2^·min^−1)^	CO_2_ Concentration	Ref.
Sm_0.2_Ce_0.8_O_2−δ−_La_0.7_Ca_0.3_CrO_3−δ_	1	900	0.17	20% CO_2_	[35]
BaFe_0.8_Ga_0.05_Ti_0.15_O_3−δ_	1	900	0.40	30% CO_2_	[36]
SrFe_0.8_Zr_0.2_O_3−δ_	1	900	0.39	10% CO_2_	[11]
La_0.1_Pr_0.2_Nd_0.2_Ba_0.2_Sr_0.2_Co_0.7_Fe_0.2_Ni_0.1_O_3−δ_	1	900	0.29	20% CO_2_	[14]
BSCFTZN	1	900	0.92	20% CO_2_	This work

**Figure A2:**
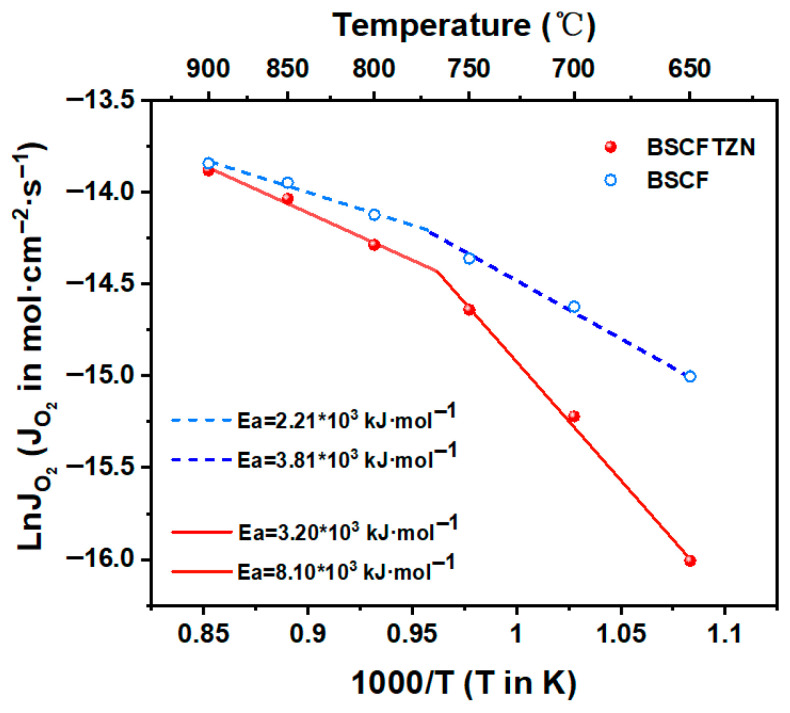
The activation energies of BSCFTZN and BSCF membranes.

## Data Availability

The data presented in this study are available upon reasonable request from the corresponding author.

## Appendix A

Figure A1 presents the Ni 2p XPS spectrum of BSCFTZN. The Ni 2p_1_/_2_ and Ni 2p_3_/_2_ peaks appear at approximately 872 and 855.5 eV, respectively. These binding energies are characteristic of Ni^3+^ species, although the presence of Ni^2+^ cannot be completely ruled out [37,38].

**Table A1:** The *δ* values of BSCF and BSCFTZN.

BSCF	Titration times and average values	δ	3 − δ
	1	0.5297	2.4701
	2	0.5312	
	3	0.5288	
	Average values	0.5299	
BSCFTZN	Titration times and average values	δ	3 − δ
	1	0.4947	2.5030
	2	0.4886	
	3	0.5078	
	Average values	0.4970	

The experiments of iodometric titration were performed under a pure N_2_ atmosphere. Approximately 0.1 g of the samples was dissolved in a 1 M HCl solution with an excess of KI, leading to the reduction of metal cations and the oxidation of I^−^ to I_2_. I_2_ was titrated with a standard Na_2_S_2_O_3_ solution using starch as the indicator.

**Table A2:** Comparison of Wt% and At% between the *theoretical* value and EDX data of the BSCFTNZ ^1^ membrane.

Elements	Wt _cal_ (%)	At _cal_ (%)	Wt _obs_ (%)	At _obs_ (%)	*X _Wt_*	*X _At_*
Ba	30.75	10.00	31.98	10.70	4.00	7.00
Sr	19.61	10.00	19.15	10.04	2.34	4.00
Co	18.72	14.20	18.55	14.45	0.91	1.76
Fe	5.00	4.00	4.76	3.92	4.80	2.00
Ta	2.43	0.60	2.66	0.68	9.5	13.33
Zr	0.79	0.60	0.86	0.78	8.86	11.66
Ni	1.23	0.60	1.56	0.67	26.82	30.00
O	21.49	60.00	20.48	58.77	4.93	2.05

^1^: Calculated according to O_3_, ignoring *δ*.

The measured EDX weight and atomic percentages for Ba, Sr, Co, Fe, and O closely match the theoretical values. In contrast, significant discrepancies were observed for Ta, Zr, and Ni, likely due to their low concentrations in the sample.
